# First Insights in NK—DC Cross-Talk and the Importance of Soluble Factors During Infection With *Aspergillus fumigatus*

**DOI:** 10.3389/fcimb.2018.00288

**Published:** 2018-08-20

**Authors:** Esther Weiss, Sabrina Ziegler, Mirjam Fliesser, Anna-Lena Schmitt, Kerstin Hünniger, Oliver Kurzai, Charles-Oliver Morton, Hermann Einsele, Juergen Loeffler

**Affiliations:** ^1^Department of Internal Medicine II, University Hospital Wuerzburg, WÜ4i, Wuerzburg, Germany; ^2^Leibniz Institute for Natural Product Research and Infection Biology—Hans Knoell Institute, Jena, Germany; ^3^Department of Microbiology and Mycology, Institute for Hygiene and Microbiology, Julius-Maximilian University, Wuerzburg, Germany; ^4^School of Science and Health, Western Sydney University, Sydney, NSW, Australia

**Keywords:** natural killer cells, dendritic cells, NK—DC cross-talk, *Aspergillus fumigatus*, soluble factors, innate immunity

## Abstract

Invasive aspergillosis (IA) is an infectious disease caused by the fungal pathogen *Aspergillus fumigatus* that mainly affects immunocompromised hosts. To investigate immune cell cross-talk during infection with *A. fumigatus*, we co-cultured natural killer (NK) cells and dendritic cells (DC) after stimulation with whole fungal structures, components of the fungal cell wall, fungal lysate or ligands for distinct fungal receptors. Both cell types showed activation after stimulation with fungal components and were able to transfer activation signals to the counterpart not stimulated cell type. Interestingly, DCs recognized a broader spectrum of fungal components and thereby initiated NK cell activation when those did not recognize fungal structures. These experiments highlighted the supportive function of DCs in NK cell activation. Furthermore, we focused on soluble DC mediated NK cell activation and showed that DCs stimulated with the TLR2/Dectin-1 ligand zymosan could maximally stimulate the expression of CD69 on NK cells. Thus, we investigated the influence of both receptors for zymosan, Dectin-1 and TLR2, which are highly expressed on DCs but show only minimal expression on NK cells. Specific focus was laid on the question whether Dectin-1 or TLR2 signaling in DCs is important for the secretion of soluble factors leading to NK cell activation. Our results show that Dectin-1 and TLR2 are negligible for NK cell activation. We conclude that besides Dectin-1 and TLR2 other receptors on DCs are able to compensate for the missing signal.

## Introduction

The saprophytic mold *Aspergillus (A.) fumigatus* causes severe infections in immunocompromised hosts especially those suffering from hematological malignancies or undergoing allogenic hematopoietic stem cell transplantation (HSCT) (Brahm and Segal, 2009). Invasive aspergillosis (IA) is the most severe form of disease where inhaled *A. fumigatus* conidia germinate and grow into deeper lung tissues and become angio-invasive.

After germination, fungal antigens are exposed and lead to substantial immunological responses mediated by immune cells of the innate and adaptive immune system (Morton et al., 2012; Jolink et al., 2013). Dendritic cells (DCs) are phagocytic cells that are a key components of the innate immune system and function as antigen presenting cells (Banchereau, 1998). Phagocytosis of pathogens activates DCs, upon they migrate into secondary lymphoid organs and present pathogenic antigens to T cells (Randolph et al., 2005). Therefore, DCs play an important role by linking innate and adaptive immune responses. DCs interact with *A. fumigatus* through the internalization receptor Dectin-1 that binds to surface ß-1,3-glucans and thereby initiates DC maturation (Mezger et al., 2008). Dectin-1 can synergize with TLR2 and this mediates enhanced cytokine production (Ferwerda et al., 2008). Signals derived from Dectin-1 and TLR2 result in the activation of the nuclear factor κB signaling pathway (Brahm and Segal, 2009; Reid et al., 2009). Furthermore, TLR9 recognizes *A. fumigatus* DNA, which induces the production of pro-inflammatory cytokines in mouse bone marrow-derived dendritic cells (BMDCs) and human plasmacytoid dendritic cells (Ramirez-Ortiz et al., 2008). Natural killer (NK) cells contribute to the innate immune system and play a major role in tumor surveillance and lysis of target cells (Waldhauer and Steinle, 2008). Besides the interaction with human cells, NK cells further participate in the control of several pathogens including viruses and fungi (Mavoungou et al., 2007; Li et al., 2013; Schmidt et al., 2013). NK-cells have been shown to interact with *Cryptococcus neoformans, Candida albicans*, and *Mucorales* (Schmidt et al., 2017). Dependent on the underlying host immune status, NK cells exerted either a beneficial or a detrimental effect on the outcome of systemic *Candida* infection in murine infection models (Quintin et al., 2014). In interaction studies of NK cells and *A. fumigatus* showed that NK cells directly interact with *A. fumigatus* through the neural cell adhesion molecule (NCAM-1, CD56) and this interaction leads to the secretion of CC chemokine ligands CCL3, 4, and 5 (Ziegler et al., 2017). After contact with *A. fumigatus*, NK cells become activated and release soluble factors such as perforin (Schmidt et al., 2011) that mediate anti-fungal activity by so far largely unknown mechanisms.

DCs and NK cells communicate in lymphoid organs and in inflamed tissue and the interactions between these cell types have been analyzed in detail in earlier studies (Fernandez et al., 1999; Cooper, 2004; Vitale et al., 2005). NK—DC cross-talk can lead to activation of both cell types and this reciprocal activation is induced by soluble and contact mediated factors (Cooper, 2004). The direct cell contact leads to high doses of cytokines at the immunological synapse, e.g., synaptical IL-12 enhances NK cell activation and cytotoxicity (Zhang et al., 2008).

Interaction between either NK cells or DCs with *A. fumigatus* have been characterized, however, the reciprocal interactions between DCs and NK cells in the presence of the fungus have not been studied before. Therefore, we firstly investigated *in vitro* NK—DC interactions in the presence of *A. fumigatus* by flow cytometry and cytokine profiling. We showed reciprocal activation of NK cells and DCs with cells that had previously been activated by co-culturing with *A. fumigatus*. Furthermore, we showed that soluble factors derived from DCs after fungal stimulation play an important role in NK cell activation.

## Materials and methods

### Cell culture

Human peripheral blood mononuclear cells (PBMCs) were isolated from healthy donors (*n* = 22) by Ficoll standard density gradient centrifugation (Biochrom AG). Monocytes were isolated according to the manufacturer's instructions (CD14 positive selection, Miltenyi Biotec). To generate monocyte-derived dendritic cells, 10 ng/ml interleukin (IL)-4 (Miltenyi Biotec) and 100 ng/ml GM-CSF (Bayer) were applied to RPMI 1640 (Invitrogen) supplemented with 10 % fetal bovine serum (FBS, Sigma Aldrich) and 120 μg/ml gentamicin (Merck) for 5 days as reported recently (Mezger et al., 2008; Tan et al., 2013; Hellmann et al., 2017). DC generation was performed in 6-well plates (BD Falcon) with a cell concentration of 2.5 × 10^6^ cells/3 ml. DC purity was confirmed by flow cytometry (Supplementary Figure 12). To preserve autologous NK cells for later NK cell isolation, 5 × 10^7^/ml PBMCs were frozen (−80°C) in FBS containing 8% dimethyl-sulfoxide (DMSO, Roth) for 5 days. After thawing, PBMC viability was 71.9 ± 0.01%. Several washing steps were performed to remove dead cells and PBMC viability (>94%) was determined by trypan blue staining (VICELL XR, Beckman Coulter). NK cells were isolated by negative selection (Miltenyi Biotec) according to the manufacturer's instructions. NK cell viability was determined by trypan blue staining and was constantly over 95 %. When DCs were stimulated first, NK cells were isolated directly on the day of co-culture. When NK cell stimulation was performed first, NK cells were pre-stimulated with 1,000 U/ml Proleukin (Novartis) overnight.

### Flow cytometry

DC generation was confirmed by staining with anti-CD14 (BD) and anti-CD1a (BD) antibodies. DCs were CD14 negative (>96%) and showed a CD1a negative (9 ± 4%) and CD1a positive (90 ± 4%) population, which are both DC populations (Cernadas et al., 2009). Anti-HLADR (BD), anti-CD80 (Miltenyi Biotec), anti-CD86 (Biolegend), anti-CCR7 (Miltenyi Biotec), and anti-CD40 (Beckman Coulter) antibodies were used to determine DC maturation by flow cytometry. NK cells were defined as NKp46^+^ and CD3^−^ cells with purity of at least 96%. NK cell gating was performed as previously described (Ziegler et al., 2017). Characterization and activation of NK cells was investigated by using the following antibodies: anti-CD69 (Miltenyi Biotec), anti-CD69 (Biolegend), anti-NKp46 (BD), anti-CD3 (BD). Isotypes were included in each experiment. TLR2 blocking was monitored by staining with a goat-anti-mouse antibody (BD) against mouse TLR2 blocking antibody.

We directly compared samples stained in HBSS + 1% FCS + 0.4% EDTA and with the addition of FcR Blocking Reagent (Miltenyi Biotec). Since no significant differences were achieved, all assays were performed without FcR Blocking Reagent (Supplementary Figure 11). Flow cytometric analyses were performed using FACSCalibur (BD-Bioscience) and data were evaluated by FlowJo Software (TreeStar). DC marker expression was displayed as mean fluorescence intensity (MFI). This was a more sensitive option to display differences between treated samples and controls, since some DC markers were already expressed to nearly 100% on unstimulated DCs while MFI still increased with stimulation (Supplementary Figures 3–5, 7). For NK cells, CD69 percent positivity was displayed as this was already used in former studies (Ziegler et al., 2017). Furthermore, we demonstrate CD69 MFI for NK cells in the Supplement (Supplementary Figures 3–6, 8, 9).

### Dectin-1 silencing

DCs were harvested at the 4th day after monocyte isolation and were resuspended in RPMI 1640 (without phenol-red) with a concentration of 1 × 10^7^ cells/ml. To 100 μl DC suspension, 6.6 μl Dectin-1 siRNA [20 μM] (SI03069780, Qiagen) or non-silencing control (1027292, Qiagen) were added and mixture was transferred into a 4-mm electroporation cuvette (Biorad). The electroporation was performed using an electroporation pulse generator (EPI 2500, Dr. L. Fischer) set to a rectangle pulse of 340 V for 10 ms. DCs were plated in RPMI 1640 + 10% FBS and fresh cytokines in 12 well plates (BD Falcon) at a cell concentration of 1 × 10^6^ cells/ml in 1 ml per well. DCs were incubated for 24 h prior to stimulation. Non-electroporated cells showed a higher survival compared to electroporated cells. Thus, all results were restricted on the direct comparison between electroporated DCs only (Dectin-1 siRNA vs. non-silencing siRNA). The efficiency of Dectin-1 silencing was monitored by real-time (RT) PCR (StepOne Plus, Thermo Fisher Scientific). Therefore, RNA was isolated from mock silenced and Dectin-1 silenced DCs by RNeasy Mini kit (Qiagen) before cDNA synthesis (First Strand cDNA Synthesis Kit, K1612, Thermo Fisher Scientific) was performed. RT-PCR was performed by using iQ™ SYBR® Green Supermix (Biorad) and either Dectin-1 specific primers (forward: CTGGTGATAGCTGTGGTCCTG; reverse primer: AAGAACCCCTGTGGTTTTGACA) or human ALAS specific primers (forward primer: GGCAGCACAGATGAATCAGA; reverse primer: CCTCCATCGGTTTTCACACT). Gene silencing of Dectin-1 was 86.38% compared to transfection control.

### TLR2 blocking

DCs were harvested at the 5th day after monocyte isolation and were treated with 10 μg/ml TLR2 blocking antibody (#mab2-mtlr2, Invivogen) or isotype control (#mabg1-ctrlm, Invivogen) for 1 h at 37°C at a cell concentration of 2 × 10^6^ cells/ml. Then, the antibody was diluted to 5 μg/ml during DC stimulation at a cell concentration of 1 × 10^6^ cells/ml. As a positive control for TLR2 blocking, cells were stained after 24 h of stimulation with a goat-anti-mouse APC (BD) antibody.

### Inactivation of *A. fumigatus* germ tubes

For germ tube generation, conidia from the *A. fumigatus* strain ATCC 46645 (in RPMI 1640) were put into a shaker for 10 h at 200 rpm and 25°C.

Germ tube inactivation was performed by incubation in 100% ethanol as previously described (Semmlinger et al., 2014).

### Cell contact dependent activation

For direct cell-to-cell contact, 16 h of co-incubation was chosen. This NK—DC co-cultivation time was used in other studies before (Krebs et al., 2012). Cell wall fractions were isolated from the mycelial cell wall of *A. fumigatus* strain CEA17 (pyrG) (Beauvais et al., 2005; Mouyna et al., 2010). The alkali insoluble (AI) cell wall fraction (working concentration 5 μg/ml) is rich in ß-1,3 glucans, chitin, galactomannans and galactosaminogalactans, whereas the alkali soluble (AS) fraction (working concentration 10 μg/ml) consists of α-1,3 glucans and galactomannans (Henry et al., 2012). A. fumigatus lysate is commercially available from Lophius Biosciences and was used at a working concentration of 1 μg/ml (for NK cell stimulation) and 5 μg/ml (for stimulation of DCs), respectively. The lysate was prepared from whole fungal cells and clarified by centrifugation in phosphate buffered saline (Lophius Biosciences). As a positive control, NK cells were stimulated with 500 IU/ml IL-15 (Immunotools) and DCs were stimulated with 1 μg/ml lipopolysaccharide (LPS, Invivogen). After stimulation of DCs or NK cells for 24 h [in 6 well plates (BD Falcon) at a cell concentration of 1 × 10^6^ cells/ml in 2 ml per well], medium was exchanged, replaced by fresh medium without any stimuli and activated DCs or NK cells were co-cultured with their unstimulated counterpart cell type using a NK to DC ratio of 1:1 [in 12 well plates (BD Falcon) at a cell concentration of 1 × 10^6^ cells/ml in 1.2 ml per well]. Following 16 h of co-incubation, the ability of the stimulated cell type to transfer activating signals to the resting, counterpart cell type was measured by flow cytometry.

### Transfer of cell culture supernatants

Dectin-1 silenced or mock silenced DCs were stimulated with inactivated *A. fumigatus* germ tubes (MOI 1), AI fraction (5 μg/ml), the Dectin-1 ligand zymosan depleted (100 μg/ml, Invivogen) and the Dectin-1/TLR2 ligand zymosan (10 μg/ml, Invivogen) for 9 h in 12 wells (BD Falcon) at a cell concentration of 1 × 106 cells/ml in 1 ml per well. Nine hours of stimulation were chosen, since DCs still showed appropriate Dectin-1 silencing rates after stimulation. When TLR2 was blocked, DCs were further stimulated with the TLR2/TLR6 stimulating agent FSL (100 ng/ml, Invivogen). FSL was used as a positive control recommended by the manufacturer (Invivogen). Stimulation of TLR2 blocked or isotype treated DCs was set for 24 h in 24 well plates (BD Falcon) at a cell concentration of 1 × 106 cells/ml in 0.4 ml per well. Extended stimulation time of TLR2 blocked cells compared to Dectin-1 silenced cells was used as DCs needed a longer incubation time for CD80 up-regulation if stimulated with the positive control FSL. After stimulation, supernatants of DCs were transferred onto resting, autologous NK cells. For NK cell incubation in supernatants derived from DCs, we used 16 h, which was adapted to our NK—DC co-culture times. NK cells were cultured in 96-well plates (BD Falcon) at a cell concentration of 1 × 10^6^ cells/ml in 0.2 ml per well. NK cell activation was measured by flow cytometry using anti-CD69 antibody.

### NK cell stimulation with cytokines

NK cells were isolated from thawed PBMCs and were stimulated with cytokines and chemokines for 16 h. Each cytokine or chemokine was used in a lower and higher concentration: IL-6 (CellGenix, 10 and 50 ng/ml), IL-10 (Miltenyi Biotec, 1 and 5 ng/ml), IP-10 (R&D, 4 and 5 ng/ml), and IL-12 (Miltenyi Biotec, 5 and 10 ng/ml). NK cells were further stimulated with a combination of all cytokines and chemokines either with the lower or higher concentration. After 16 h, NK cell activation was measured by flow cytometry using an anti-CD69 antibody.

### Quantification of secreted proteins

Supernatants of DCs were analyzed by ProcartaPlex® Multiplex Immunoassay (eBioscience) for the following cytokines: interleukin (IL)-1α, IL-1β, IL-2, IL-6, IL-8, IL-10, IL-12p70, IL-15, IL-18, TNF-α, MIP-3α, IP-10, and IFN-γ (Bio-Plex 200 System, Bio-Rad). We avoided freeze—thawing cycles of supernatants and analyzed supernatants after storing for 10 weeks at −20°C. Cytokine degradation was minimal and there were no significant differences between short—term (10 weeks) and long—term (56 weeks) storage when supernatants were compared by two-way ANOVA (Supplementary Figure 10).

### Statistical analysis

Data analyses were performed by using a *t*-test or ANOVA + Bonferroni correction. Statistics were calculated using GraphPad Prism 7 software.

## Results

Direct cell-to-cell contact was analyzed by co-culture of previously stimulated cells with the unstimulated counterpart cell type. For stimulation of cells, alkali insoluble (AI) and alkali soluble (AS) mycelium cell wall fractions as well as *A. fumigatus* (AF) lysate were used. AI is rich in ß-1,3 glucans, chitin, galactomannans and galactosaminogalactans, whereas AS consists of α-1,3 glucans and galactomannans (Henry et al., 2012). AF lysate is a whole cell lysate and in contrast to the cell wall fractions also includes intracellular stimulants.

**Figure 1:**
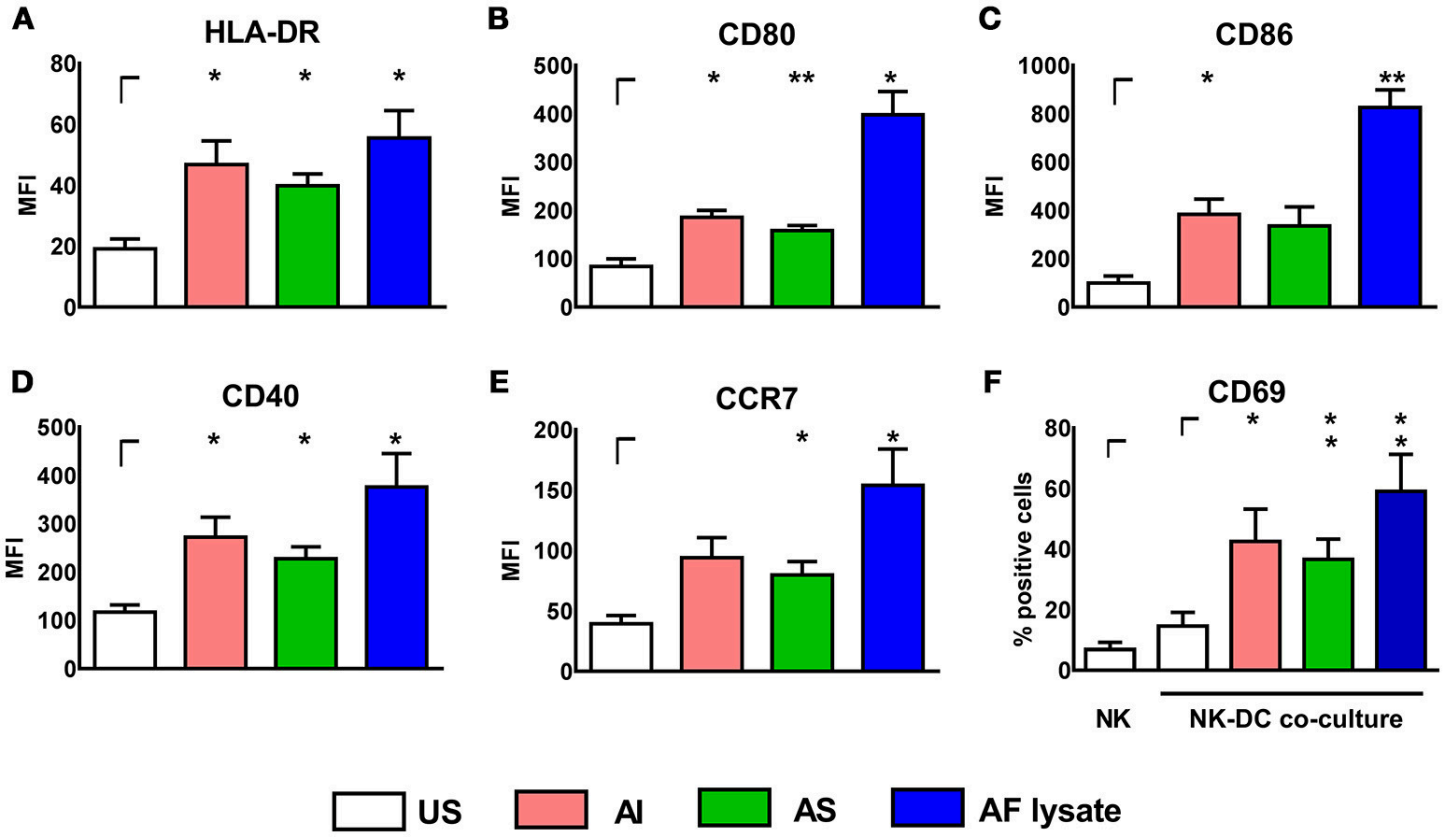
*A. fumigatus* stimulated DCs can activate autologous, resting NK cells. DCs were either left untreated (US) or were stimulated with soluble (AS, 10 μg/ml) and insoluble (AI, 5 μg/ml) cell wall fractions or *A. fumigatus* lysate (AF lysate, 5 μg/ml) for 24 h. Flow cytometry was performed to measure the mean fluorescence intensity (MFI) of the maturation markers **(A)** HLA-DR, **(B)** CD80, **(C)** CD86, **(D)** CD40, and **(E)** CCR7. **(F)** NK cells were left untreated (NK) or co-cultured with previously AI, AS, AF lysate stimulated or unstimulated DCs (US). NK cell activation was measured by the percentage of CD69 expressing NK cells. Data are represented as mean + SEM of three independent experiments. Student's test was performed and significant differences are marked with an asterisk (*p < 0.05, **p < 0.01).

All three stimulants induced a significant up-regulation of the DC maturation markers HLA-DR, CD80, CD86, CD40, and CCR7, with AF lysate showing the most prominent effect on DC maturation (Figures 1A–E). After determination of DC maturation, the supernatants containing the soluble stimulants were removed by medium exchange and DCs were co-cultured with autologous, resting NK cells for 16 h (Figure 1F). Interestingly, NK cells upregulated the activation marker CD69 after co-culture with stimulated DCs, whereas unstimulated DCs did not induce NK cell activation (Figure 1F).

**Figure 2:**
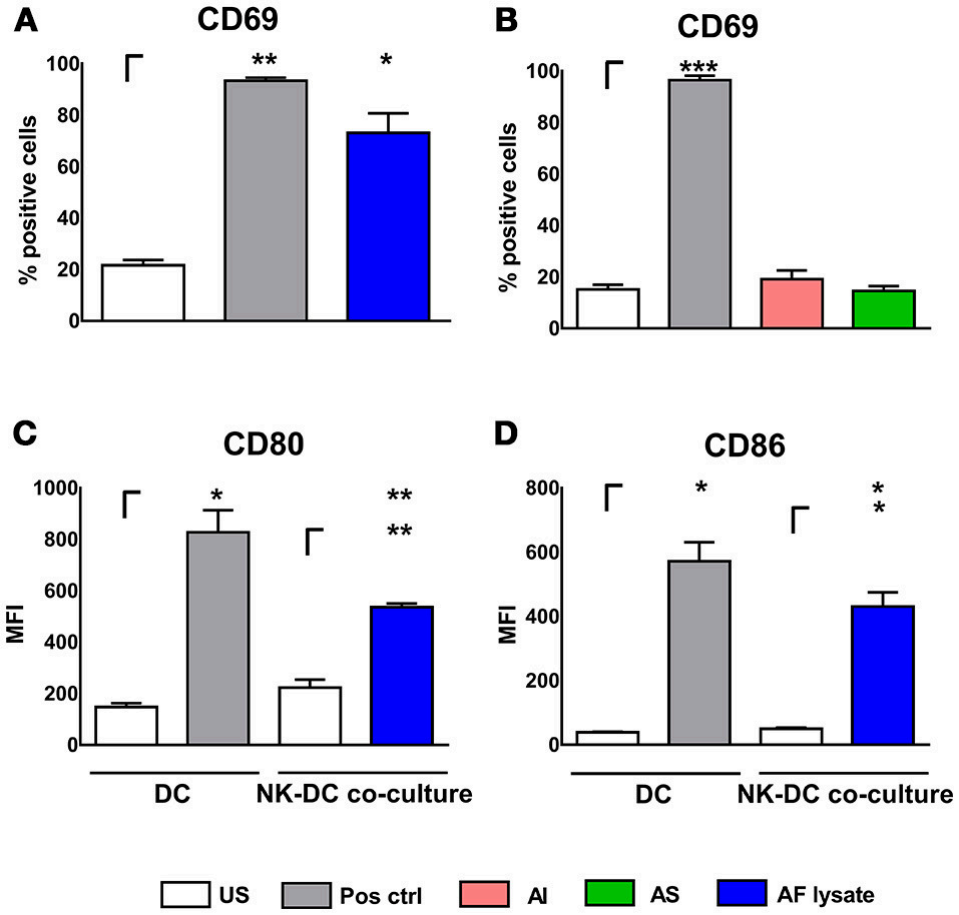
NK cell activation after stimulation with fungal components and NK—DC co-culture. NK cells were either left untreated (NK US), or were stimulated with **(A)** *A. fumigatus* lysate (NK AF lysate, 1 μg/ml) and with positive control (Pos. ctrl., 500 U/ml IL-15) or **(B)** with alkali insoluble cell wall fraction (AI, 5 μg/ml), alkali soluble cell wall fraction (AS, 10 μg/ml) or positive control (Pos. ctrl, 500 U/ml IL-15) for 24 h. NK cell activation was quantified by the expression of CD69 positive NK cells. **(C,D)** DCs were either left untreated (US) or were co-cultured with autologous, unstimulated (NK US) or pre-stimulated (NK AF lysate) NK cells for 16 h. As a positive control, DCs were stimulated with 1 μg/ml LPS (Pos. ctrl.). DC maturation was investigated by the measurement of the mean fluorescence intensity (MFI) of **(C)** CD80 and **(D)** CD86 costimulatory molecules. Data are represented as mean + SEM of three independent experiments. A student's *t*-test was performed and significant differences are marked with an asterisk (*p < 0.05, **p < 0.01, ***p < 0.001).

Experiments were also performed vice versa by stimulating NK cells with AI, AS, and AF lysate for 24 h. NK cells increased CD69 expression after stimulation with AF lysate (Figure 2A) but showed no significant up-regulation after treatment with AI or AS (Figure 2B) even when stimulated with concentrations up to 20 μg/ml (Supplementary Figure 1). To investigate whether activated NK cells can transfer stimulating signals to immature DCs, NK cells were treated either with AF lysate or plain medium for 24 h (Figure 2A). NK cell supernatants were removed and NK cells were resuspended in fresh medium before NK—DC co-culture was set for 16 h. DCs displayed a significant up-regulation of the maturation markers CD80 and CD86 after co-cultivation with AF lysate stimulated NK cells but not after co-culture with unstimulated NK cells (Figures 2C,D), indicating that only activated NK cells can induce DC maturation.

These experiments firstly showed that DCs and NK cells interact during infection with *A. fumigatus* and that those interactions lead to cell maturation or activation. DCs were stimulated by all three stimulants (Figures 1A–E) while NK cells exhibited cell activation only after stimulation with AF lysate (Figures 2A,B). However, NK cells were successfully stimulated by DCs that were treated with stimulants that do not activate NK cells in single culture, implying that DCs are relevant cells for effective activation of NK cells during infection with *A. fumigatus*.

**Figure 3:**
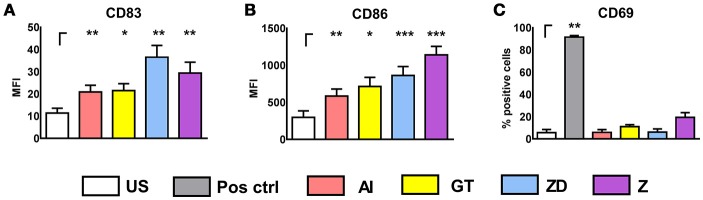
Stimulation of DCs and NK cells with cell wall fraction, inactivated germ tubes, zymosan depleted, and zymosan. DCs were left untreated (DC US) or were stimulated with alkali insoluble cell wall fraction (AI, 5 μg/ml), inactivated *A. fumgiatus* germ tubes (GT, MOI 1), zymosan depleted (ZD, 100 μg/ml), or zymosan (Z, 10 μg/ml) for 9 h. DC maturation was analyzed by the measurement of the mean fluorescence intensity (MFI) of **(A)** CD83 and **(B)** CD86. Data are represented as mean + SEM of *n* = 6 independent experiments. **(C)** NK cells were left untreated (NK) or were stimulated with either the positive control (IL-15, 500 U/ml), AI (5 μg/ml), GT (MOI 1), ZD (100 μg/ml), or Z (10 μg/ml) for 16 h. NK cell activation was investigated by measuring the percentage of CD69 expression with flow cytometry. Data are represented as mean + SEM **(C)** *n* = 3 independent experiments. A student's *t*-test was performed and significant differences are marked with an asterisk (*p < 0.05, **p < 0.01, ***p < 0.001).

Human NK cells express Dectin-1 and TLR2 only minimally on their surface (Ziegler et al., 2017) and therefore stimulation with TLR2 and Dectin-1 ligands induce DC maturation (Figures 3A,B) but no NK cell activation (Figure 3C, Supplementary Figure 2).

To investigate whether Dectin-1 or TLR2 signaling in DCs may be responsible for NK cell activation by the secretion of stimulating cytokines, we transferred supernatants from Dectin-1 and TLR2 silenced or blocked DC stimulations onto resting, autologous NK cells. For stimulation we used different components containing ligands for Dectin-1 and TLR2. Dectin-1 recognizes the highly abundant ß-glucans in the cell wall of *A. fumigatus* (Brown et al., 2002) and TLR2 binds to fungal conidia and hyphae (Chai et al., 2009). However, the exact ligand for TLR2 on *A. fumigatus* remains unknown until now.

**Figure 4:**
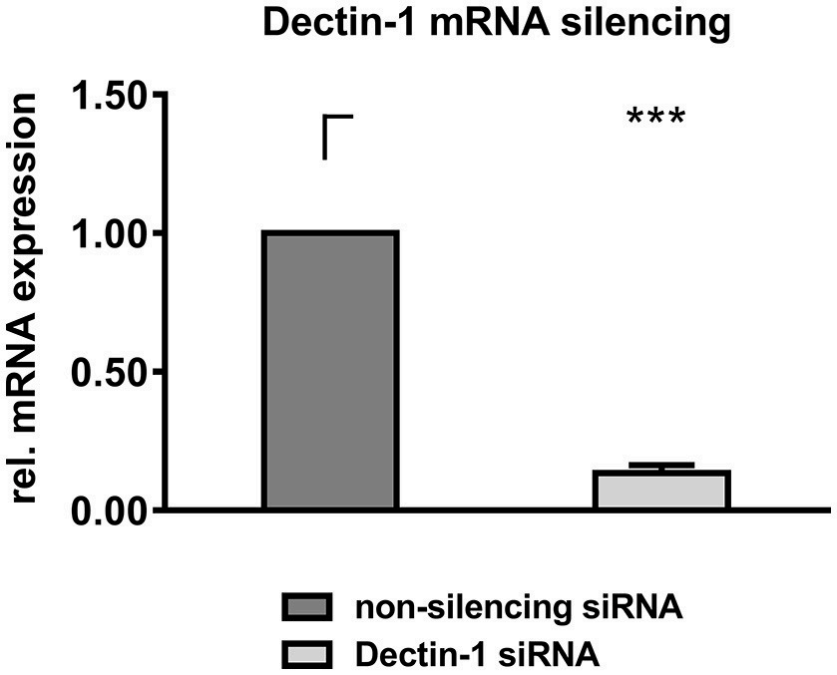
Dectin-1 silencing. DCs were electroporated with Dectin-1 siRNA or non-silencing siRNA and were cultured for 24 h at 37°C. RNA was isolated from 1 × 10^6^ cells and was converted to cDNA. RT-PCR was performed to quantify Dectin-1 downregulation on mRNA level. Dectin-1 mRNA expression level of Dectin-1 silenced DCs was calculated compared to a non-silencing control. Data are represented as mean + SEM of *n* = 5 independent experiments. A student's *t*-test was performed and significant differences are marked with an asterisk (***p < 0.001).

Mock silenced DCs and Dectin-1 silenced DCs were stimulated with AI, inactivated *A. fumigatus* germ tubes (GT), zymosan depleted (ZD), and zymosan (Z) for 9 h before DC supernatants were transferred onto autologous, resting NK cells for 16 h. Gene silencing of Dectin-1 was 86.38% compared to transfection control (Figure 4).

**Figure 5:**
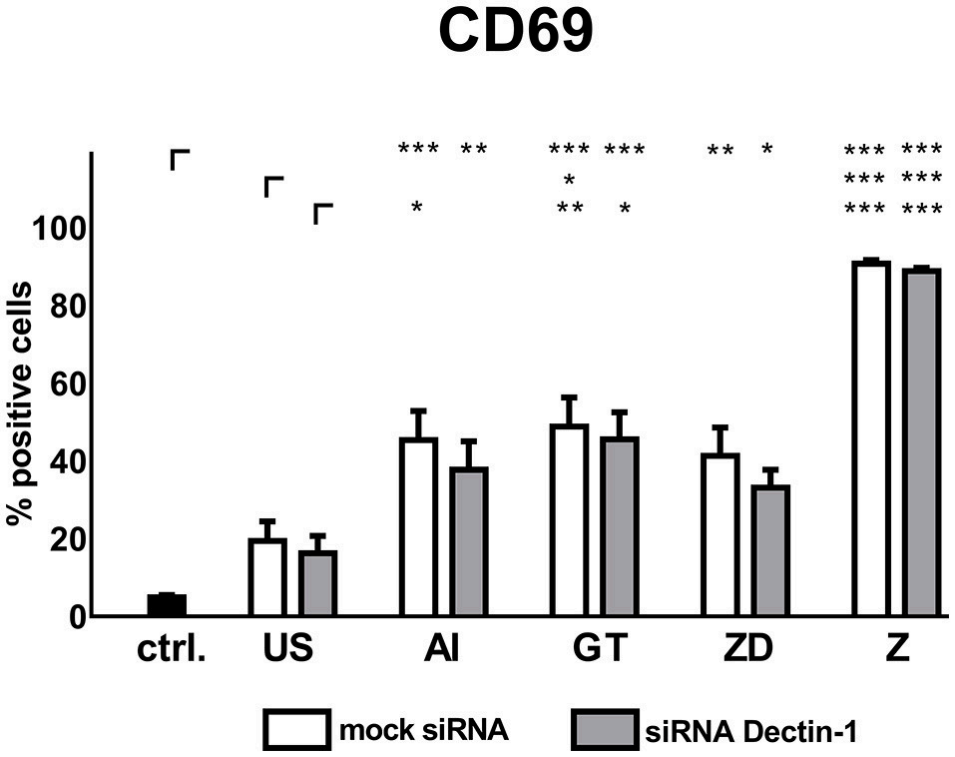
Soluble factors derived from Dectin-1 silenced and mock silenced DCs activate autologous NK cells. Dectin-1 silenced or mock silenced DCs were stimulated with the alkali insoluble cell wall fraction (AI, 5 μg/ml), inactivated *A. fumigatus* germ tubes (GT, MOI 1), zymosan depleted (ZD, 100 μg/ml), zymosan (Z, 10 μg/ml), or were left untreated (US) for 9 h. Soluble factors from DC stimulation were transferred onto resting, autologous NK cells for 16 h. NK cell activation was assessed by the expression of CD69. Data are represented as mean + SEM of *n* = 5 independent experiments. ANOVA + Bonferroni correction was performed and significant differences are marked with an asterisk (*p < 0.05, **p < 0.01, ***p < 0.001).

Supernatants from unstimulated DCs enhanced CD69 expression (mock: 19.37%, siRNA: 16.27%) compared to NK cells treated with blank medium (4.90%, Figure 5). Mock silenced DC supernatants of AI, GT or ZD stimulations induced a moderate CD69 expression (41.26–48.84%, Figure 5) whereas supernatants derived from stimulation with Z boosted NK cell activation (90.76%, Figure 5).

Supernatants of mock silenced DCs induced higher CD69 expression (41.3 %) compared to Dectin-1 silenced DCs (33.1%) when stimulated with the Dectin-1 ligand ZD (Figure 5).

Soluble factors from DCs stimulated with AI showed a trend toward decreased NK cell activation compared to mock silenced controls treated with AI (Figure 5). However, Dectin-1 silencing could not significantly reduce NK cell activation, concluding that there might be other DC receptors besides Dectin-1 with more impact on NK cell activation.

**Figure 6:**
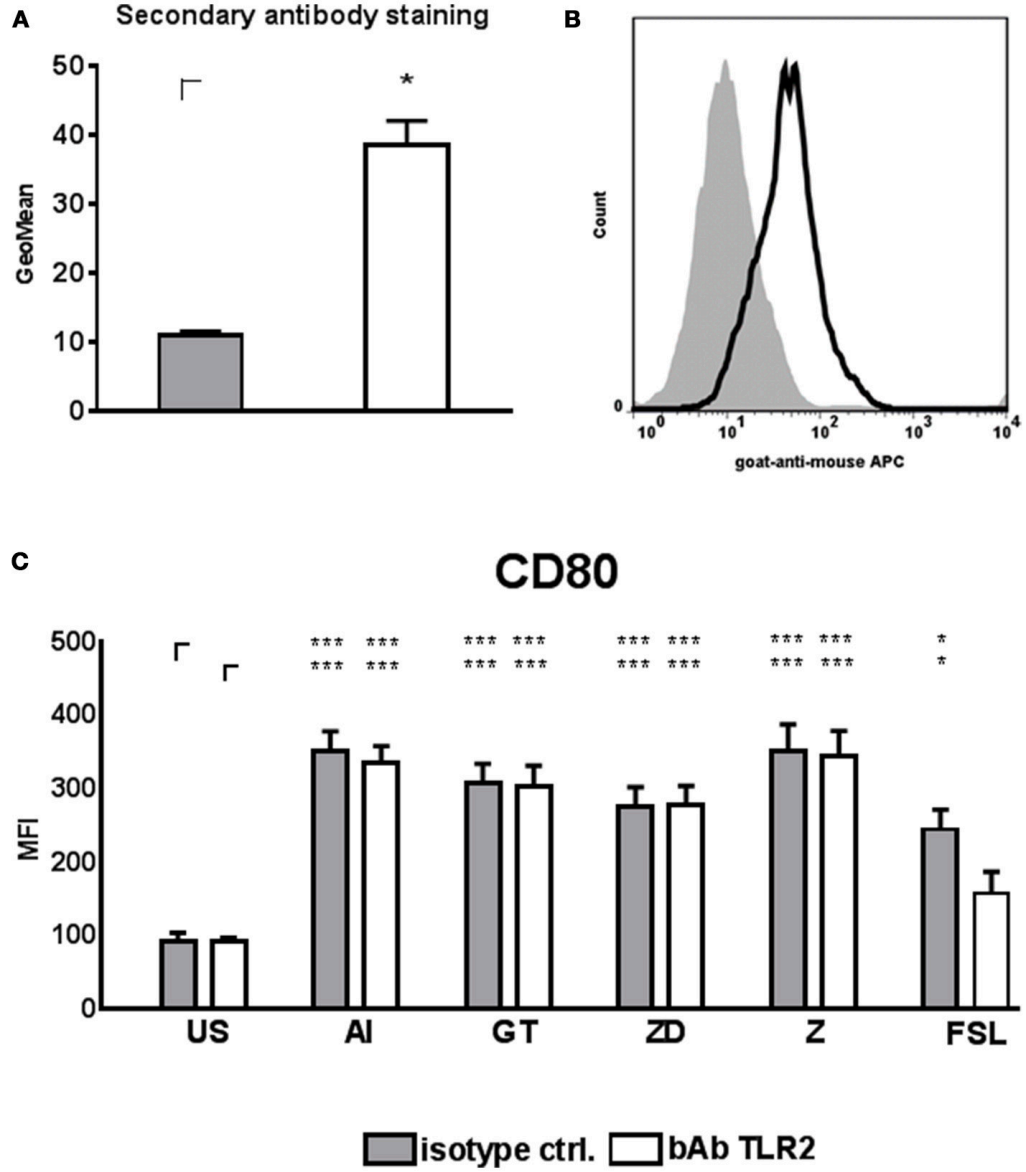
TLR2 blocking and DC stimulation. DCs were treated with TLR2 blocking antibody (10 μg/ml) or isotype control at a cell concentration of 2 × 10^6^ cells/ml for 1 h at 37°C. DCs were diluted to 1 × 10^6^ cells/ml with medium and stimulated with the alkali insoluble cell wall fraction (AI, 5 μg/ml), inactivated *A. fumigatus* germ tubes (GT, MOI 1), zymosan depleted (ZD, 100 μg/ml), zymosan (Z, 10 μg/ml), FSL (100 ng/ml), or were left untreated (US) for 24 h. **(A)** DCs treated with TLR2 blocking antibody or isotype control were stained with goat anti-mouse secondary antibody. Geo Mean of fluorescence was analyzed from *n* = 3 independent experiments. A student's *t*-test was performed and significant differences are marked with an asterisk (*p < 0.05). **(B)** Fluorescence histogram of secondary antibody staining of one out of 3 independent experiments is shown. **(C)** CD80 mean fluorescence intensity (MFI). Data are represented as mean + SEM of *n* = 5 independent experiments. ANOVA + Bonferroni correction was performed and significant differences are marked with an asterisk (*p < 0.05, ***p < 0.001).

Thus, we next tested whether TLR2 signaling in DCs is important for DC mediated NK cell activation. DCs were treated with TLR2 blocking antibody (bAb) or isotype control for 1 h before DCs were stimulated with AI, GT, ZD, and Z. FSL, a synthetic diacylated lipoprotein derived from *Mycoplasma salivarium* (Shibata et al., 2000), is recognized by TLR2/TLR6 heterodimers (Okusawa et al., 2004) and was used as a positive control as suggested by the manufacturer. DCs were stimulated for 24 h and DC maturation was measured by flow cytometry using anti-CD80 antibody (Figure 6C). As a control for successful TLR2 blocking bAb and control treated DCs were stained after 24 h with goat-anti-mouse secondary antibody (Figures 6A,B).

The TLR2/6 ligand FSL only induced significant CD80 up-regulation in DCs treated with isotype control but not with TLR2 bAb, confirming successful TLR2 blocking (Figure 6C). However, TLR2 blocking had no influence on CD80 up-regulation on DCs stimulated with AI, GT, ZD and Z compared to unstimulated controls (Figure 6C).

**Figure 7:**
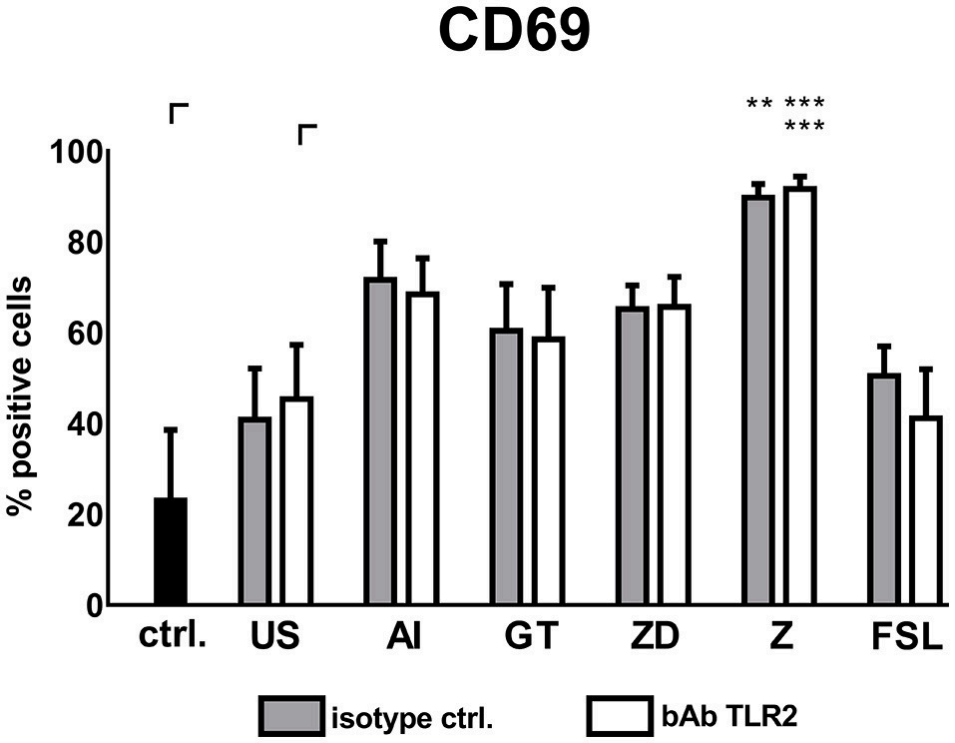
Soluble factors derived from TLR2 blocked DCs activate autologous NK cells. DCs treated with TLR2 blocking antibody (10 μg/ml) or isotype control were stimulated with the alkali insoluble cell wall fraction (AI, 5 μg/ml), inactivated *A. fumigatus* germ tubes (GT, MOI 1), zymosan depleted (ZD, 100 μg/ml), zymosan (Z, 10 μg/ml), FSL (100 ng/ml), or were left untreated (US) for 24 h. Soluble factors from DC stimulation were transferred onto resting, autologous NK cells for 16 h. NK cell activation was assessed by the expression of CD69. Untreated NK cells were used as a control (ctrl.). Data are represented as mean + SEM of *n* = 4 independent experiments. ANOVA + Bonferroni correction was performed and significant differences are marked with an asterisk (**p < 0.01, ***p < 0.001).

FSL treated DCs induced lower NK cell up-regulation when TLR2 was blocked compared to control treated DCs (Figure 7). When supernatants of TLR2 blocked DCs treated with Z were transferred onto NK cells, NK cells significantly up-regulated CD69 expression, however, bAb and isotype treated DC supernatants showed no difference in the induction of NK cell activation (Figure 7).

Taken together, neither Dectin-1 nor TLR2 inhibition could prevent DC mediated NK cell activation when we used cell wall fraction (AI) or whole fungal structures (GT) for stimulation.

**Figure 8:**
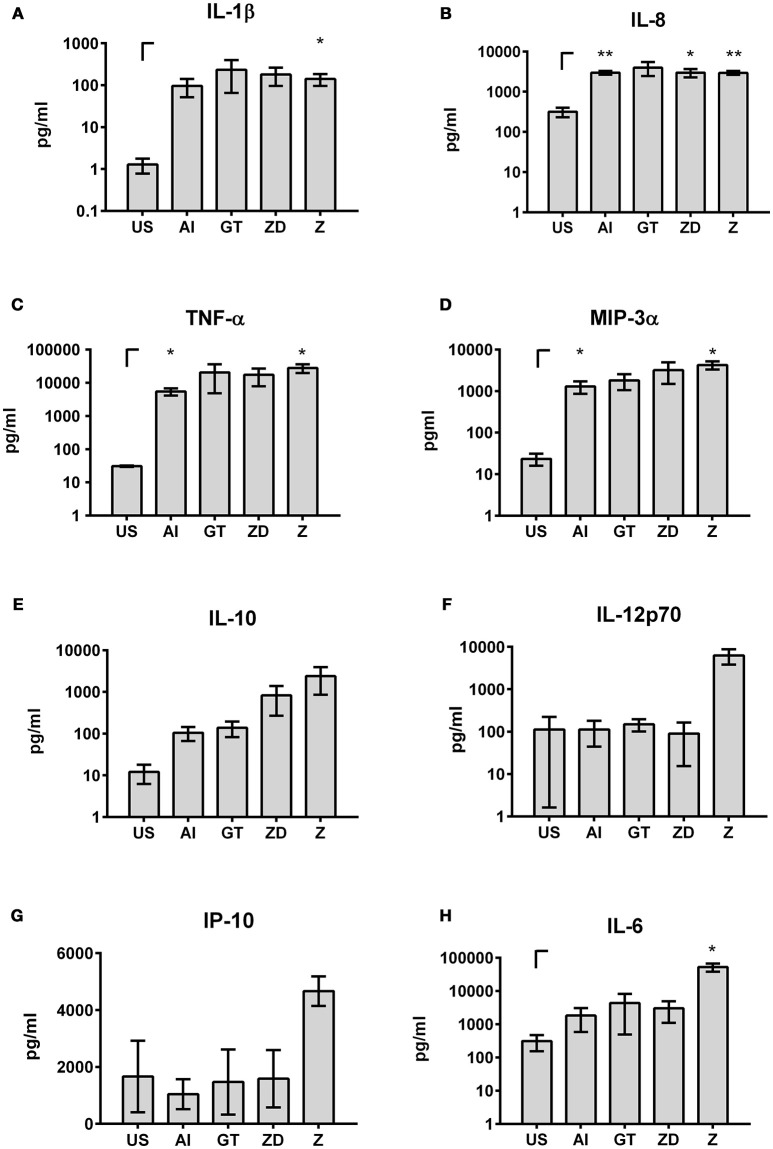
Cytokines and chemokines in DC supernatants. DCs were stimulated with the alkali insoluble cell wall fraction (AI, 5 μg/ml), inactivated *A. fumigatus* germ tubes (GT, MOI 1), zymosan depleted (ZD, 100 μg/ml), zymosan (Z, 10 μg/ml), or were left untreated (US) for 9 h. IL-1β **(A)**, IL-8 **(B)**, TNF-α **(C)**, MIP-3α **(D)**, IL-10 **(E)**, IL-12p70 **(F)**, IP-10 **(G)**, and IL-6 **(H)** cytokine levels were analyzed by multiplex immunoassay. Data are represented as mean ± SEM of *n* = 3 independent experiments. A student's *t*-test was performed to compare treated samples (AI, GT, ZD, Z) with unstimulated (US) samples. Significant differences are marked with an asterisk (*p < 0.05, **p < 0.01).

Transfer of soluble factors derived from DCs to NK cells resulted in specific NK cell activation depending on the DC stimulus that was used. Here, stimulation with Z yielded in the highest NK cell activation (Figures 5, 7). To analyze which soluble factors are secreted after stimulation with Z, several cytokines and chemokines which play a role in general immune reactions (IFN-γ, IL-6, IL-8, IL-10, IL-1α, IL-1β, MIP-3α, TNF-α) or are specifically important for NK cell activation (IL-2, IL-12p70, IL-15, IL-18, IP-10) were analyzed by multiplex immunoassay (Figure 8).

Secretion of IFN-γ, IL-2, IL-15, IL-18, and IL-1α was weak or not detectable after stimulation of DCs with the different stimuli (data not shown).

In contrast, high levels of IL-1β, IL-8, TNF-α, and MIP-3α were detected after all stimulations (IL-1β: 96.42–232.77 pg/ml; IL-8: 2.95–3.97 ng/ml; TNF-α: 5.5–28.03 ng/ml; MIP-3α: 1.29–4.24 ng/ml, Figures 8A–D). IL-6 and IL-10 were highly secreted after all stimulations but showed the highest secretion after treatment with Z (Figures 8E,H). Furthermore, stimulation with Z boosted the secretion of IL-12p70 and Interferon-γ-inducible protein (IP)-10 (Figures 8F,G). These results showed that the stimulating properties of Z are beneficial for cytokine secretion and NK cell activation.

**Figure 9:**
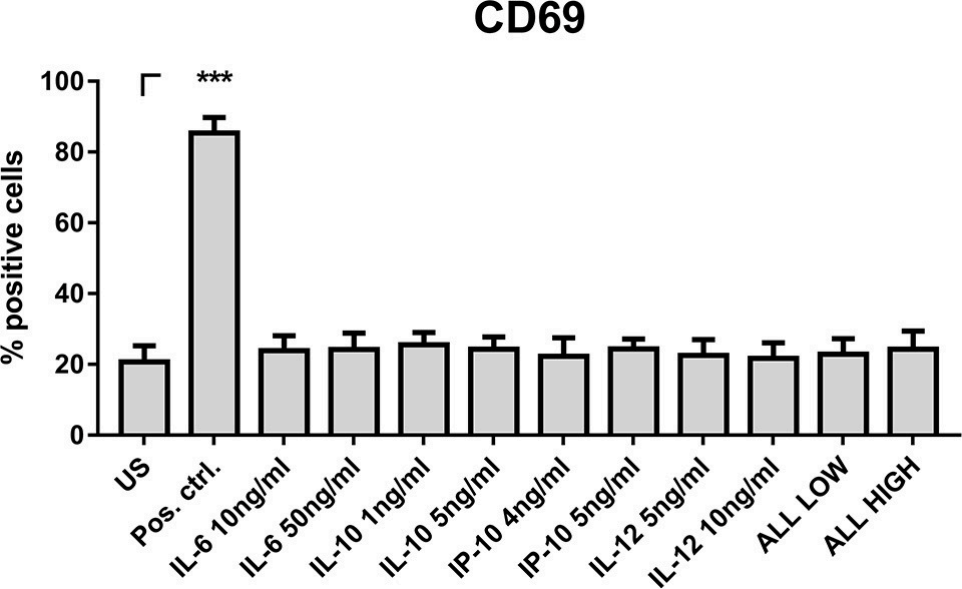
NK cell stimulation with cytokines and chemokines. NK cells were isolated from thawed PBMCs and were stimulated with IL-6 (10 and 50 ng/ml), IL-10 (1 and 5 ng/ml), IP-10 (4 and 5 ng/ml), IL-12 (5 and 10 ng/ml) or the combination of all cytokines and chemokines in low or high concentration, respectively. After 16 h, NK cell activation was analyzed by flow cytometry. Data are represented as mean + SEM of *n* = 3 independent experiments. A student's *t*-test was performed to compare treated samples with unstimulated (US) samples. Significant differences are marked with an asterisk (***p < 0.001).

To test whether the measured cytokines and chemokines after DC stimulation are sufficient to induce CD69 expression by NK cells, we thawed PBMCs, isolated NK cells and stimulated those with cytokines and chemokines (Figure 9). We specifically focused on cytokines and chemokines showing the highest concentrations after stimulation with Z (Figures 8E–H), since Z stimulated DC supernatants induced the highest NK cell activation (Figures 5, 7) and therefore were the most prominent candidates containing important cytokines and chemokines in NK—DC cross-talk.

Thus, NK cells were stimulated with a lower and a higher concentration of IL-6, IL-12, IP-10, and IL-10 within the measured range of secreted cytokines and chemokines after DC stimulation with Z (Figures 8E–H). However, only IL-15 could induce such a high CD69 expression on NK cells measured with Z stimulated DC supernatants before (Figure 9). In consequence, the cytokines and chemokines tested alone or in combination were not responsible for CD69 expression.

## Discussion

Stimulation with antigen-pulsed donor DCs can enhance the proliferation status of PBMCs derived from stem cell recipients (Grazziutti et al., 2001) and furthermore induce a protective Th1 response (Bozza et al., 2003). In addition, NK cell counts and reconstitution time inversely correlate with a higher risk of developing mold infections after stem cell transplantation showing that NK cells might have a protective effect against *A. fumigatus* (Stuehler et al., 2015).

NK—DC cross-talk may be beneficial against pathogens in several ways. Firstly, it has been shown that TLR-activated DCs can induce IFN-γ secretion by NK cells (Kamath et al., 2005), which is important for the stimulation of further cell types such as alveolar macrophages to phagocytose fungal spores (Park et al., 2009).

Secondly, NK cell killing of pathogens or pathogen infected cells may be directly enhanced by DCs. For example, CXCL10 released from DCs infected with *Mycobacterium tuberculosis* enhanced NK cell migration to sites of infection where they destroyed *Mycobacterium* infected cells (Lande et al., 2003).

To investigate if and how dendritic cell transfers may modulate the NK immune response against *A. fumigatus*, we investigated the NK—DC cross-talk *in vitro* after interaction with *A. fumigatus* and other stimulating agents. A major focus was put on the fungal receptors Dectin-1 and TLR2, which are known to be expressed on DCs but show very low expression on NK cells.

We initially analyzed whether NK cells and DCs can transfer activation signals during cell-to-cell contact. Indeed, both cell types showed activation or maturation after stimulation with *A. fumigatus* lysate and therefore can transfer stimulating signals to autologous, resting or immature cells, showing that NK cells and DCs can communicate in the immune response against *A. fumigatus*.

Interestingly, DCs furthermore recognized cell wall fractions of *A. fumigatus* that contain the Dectin-1 specific ligand ß-glucan and could activate NK cells even when those were not activated by the stimulus itself. Since NK cells rarely express Dectin-1, TLR2, or TLR4 on their surface (Ziegler et al., 2017) these experiments supported our hypothesis that DCs have a substantial supporting role in NK cell activation which may also play a role *in vivo* during infection with *A. fumigatus*.

TLR2, TLR4, Dectin-1, and Dectin-2 are important fungal recognition receptors on DCs (Netea et al., 2003; Steele et al., 2005; Loures et al., 2015). While *A. fumigatus* conidia and hyphae modulate TLR2 and TLR4 signaling (Netea et al., 2003), galactomannan is specifically bound by Dectin-2 (Reedy et al., 2016) and ß-1,3 glucans are recognized by Dectin-1 (Brown et al., 2002).

To analyze the impact of DC mediated Dectin-1 signaling on NK cells, we silenced Dectin-1 and stimulated Dectin-1 silenced and mock silenced DCs with components rich in ligands for Dectin-1. ß-glucan is a component of the fungal cell wall and therefore is represented in cell wall fractions, inactivated germ tubes and yeast-derived cell wall preparations of zymosan. Hot alkali treatment of zymosan results in the removal of TLR2 stimulating agents and therefore this depleted zymosan is exclusively stimulating Dectin-1. However, Dectin-1 silenced DCs stimulated with ZD were still able to up-regulate CD69 expression on NK cells, assuming that only few amounts of unsilenced receptors are sufficient for DC maturation. We noticed no great difference in the induction of NK cell activation between stimulated DCs treated with Dectin-1 or mock siRNA, concluding that Dectin-1 has no important impact on DC mediated NK cell activation.

We next tested the influence of TLR2 on DC mediated NK cell activation. FSL was used as a positive control recommended by the manufacturer, however, there is no exclusive TLR2 ligand known and FSL also stimulates TLR6. We detected less DC maturation after FSL stimulation when TLR2 was blocked on DCs. Other stimulants showed no difference in inducing DC maturation and NK cell activation after TLR2 blocking compared to isotype treatment.

Thus, we conclude that neither Dectin-1 nor TLR2 are important for DC-mediated NK cell activation or that other functional receptors are able to compensate function of these receptors. Redundancy of fungal receptor function was also hypothesized in the paper from Chai et al., in which Dectin-1 polymorphism resulted in less secretion of TNF-α and IL-6 from PBMCs but had no influence on cytokine secretion in monocyte-derived macrophages, claiming that these cell types can compensate lost Dectin-1 signaling by other receptors (Chai et al., 2011). It has been shown that Dectin-1 and TLR2 function together to mediate macrophage activation by mycobacteria (Yadav and Schorey, 2006). Thus, future experiments are required to analyze effects of simultaneous blocking of Dectin-1 and TLR2 on NK cell activation.

DCs secreted intermediate to high amounts of IL-1β, TNF-α, and MIP-3α which are cytokines secreted in Dectin-1 signaling (Steele et al., 2005; Kock et al., 2011). Furthermore, it has been shown that TNF-α is an important cytokine secreted after Dectin-2 encounter (Loures et al., 2015).

Interestingly, zymosan stimulation boosted NK cell activation the most. We detected specific zymosan induced cytokines which may play an important role for NK cell activation, namely IL-12p70, IL-6, IL-10, and IP-10. While IL-6 and IL-10 are general pro- and anti-inflammatory cytokines (Turner et al., 2014) IL-12p70 and IP-10 have been described as inducers of NK cell activation which are at least partially dependent on TLR2 signaling (Lehmann et al., 2001; Roberts et al., 2014; Liu et al., 2016).

Therefore, we tested whether physiological concentrations of IL-6, IL-10, IL-12, or IP-10 can induce CD69 expression like supernatants of DCs stimulated with Z. Even when used in combination, IL-6, IL-12, IL-10, or IP-10 could not induce CD69 expression on NK cells. These experiments lead to the conclusion that there are additional important soluble factors that induce NK cell activation after DC stimulation with TLR2/Dectin-1 ligands.

In summary, this is the first study that gives insight into the soluble factors mediating NK—DC cross-talk during infection with *A. fumigatus in vitro*. Future studies are required to deeper characterize NK—DC cross-talk regarding the importance of cellular and soluble factors.

## Ethics statement

Use of whole blood specimens from healthy volunteers was approved by the University Hospital of Wuerzburg Ethical Committee (#302/15). Written informed consent was obtained and data analysis was performed anonymously.

## Author contributions

EW developed concepts, designed the overall study, performed experiments, performed data analyses and wrote the manuscript. SZ and MF developed concepts and revised the manuscript. A-LS performed experiments and helped with designing the study. KH performed the multiplex ELISA and revised the manuscript. OK, C-OM, and JL provided discussions and contributed to the manuscript. HE and JL developed concepts, supervised the study and wrote the manuscript.

### Conflict of interest statement

The authors declare that the research was conducted in the absence of any commercial or financial relationships that could be construed as a potential conflict of interest.
