# Supplementary material for: First Insights in NK—DC Cross-Talk and the Importance of Soluble Factors During Infection With Aspergillus fumigatus
Source: Front Cell Infect Microbiol. 2018 Aug 20;8:288. doi: 10.3389/fcimb.2018.00288 (PMC6110135; doi:10.3389/fcimb.2018.00288)
Supplement: Supplementary file 1 [file Data_Sheet_1.PDF]

Supplementary Figure 1:

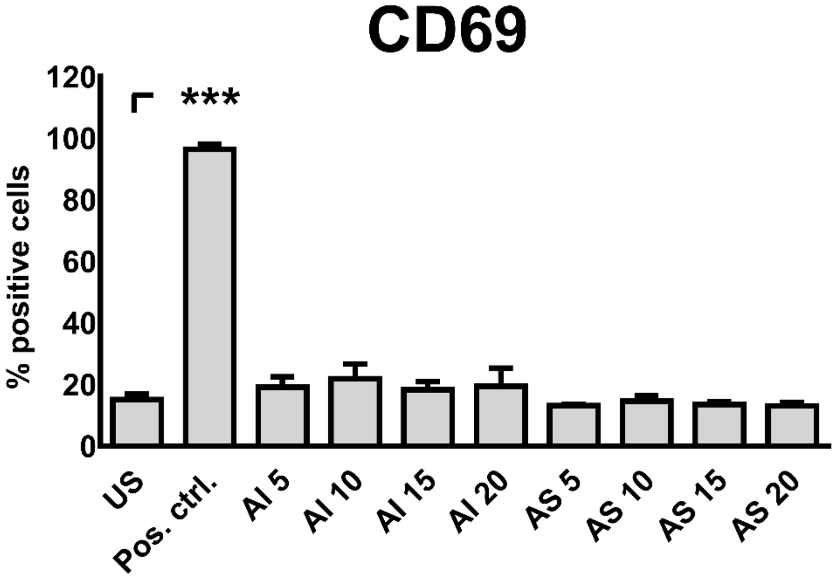

NK cell activation after stimulation with *A. fumigatus* cell wall fractions. NK cells were isolated by negative isolation (Miltenyi Biotec) from fresh PBMCs and were pre-stimulated with 1000 U/ml Proleukin overnight. NK cells were treated with IL-15 (Pos. ctrl., 500 U/ml), alkali insoluble cell wall fraction (AI, 5 - 20 µg/ml), alkali soluble cell wall fraction (AS, 5 - 20 µg/ml) or were left untreated (US). Data are represented as mean + SEM of n = 3 independent experiments. A student’s t-test was performed and significant differences are marked with an asterisk (*** $p < 0.001$).

Supplementary Figure 2:

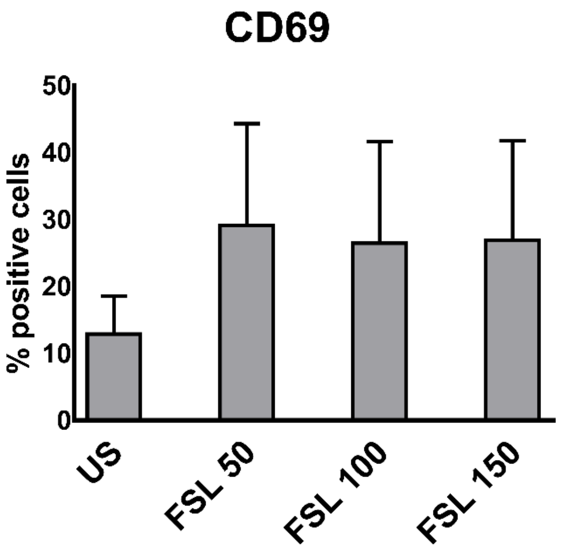

NK cell activation after stimulation with FSL. NK cells were isolated by negative isolation (Miltenyi Biotec) from thawed PBMCs and were stimulated with FSL ligand (50 ng/ml, 100 ng/ml or 150 ng/ml) for 16 h. Data are represented as mean + SEM of n = 3 independent experiments. A student's t-test was performed but no significant differences were detected.

Supplementary Figure 3:

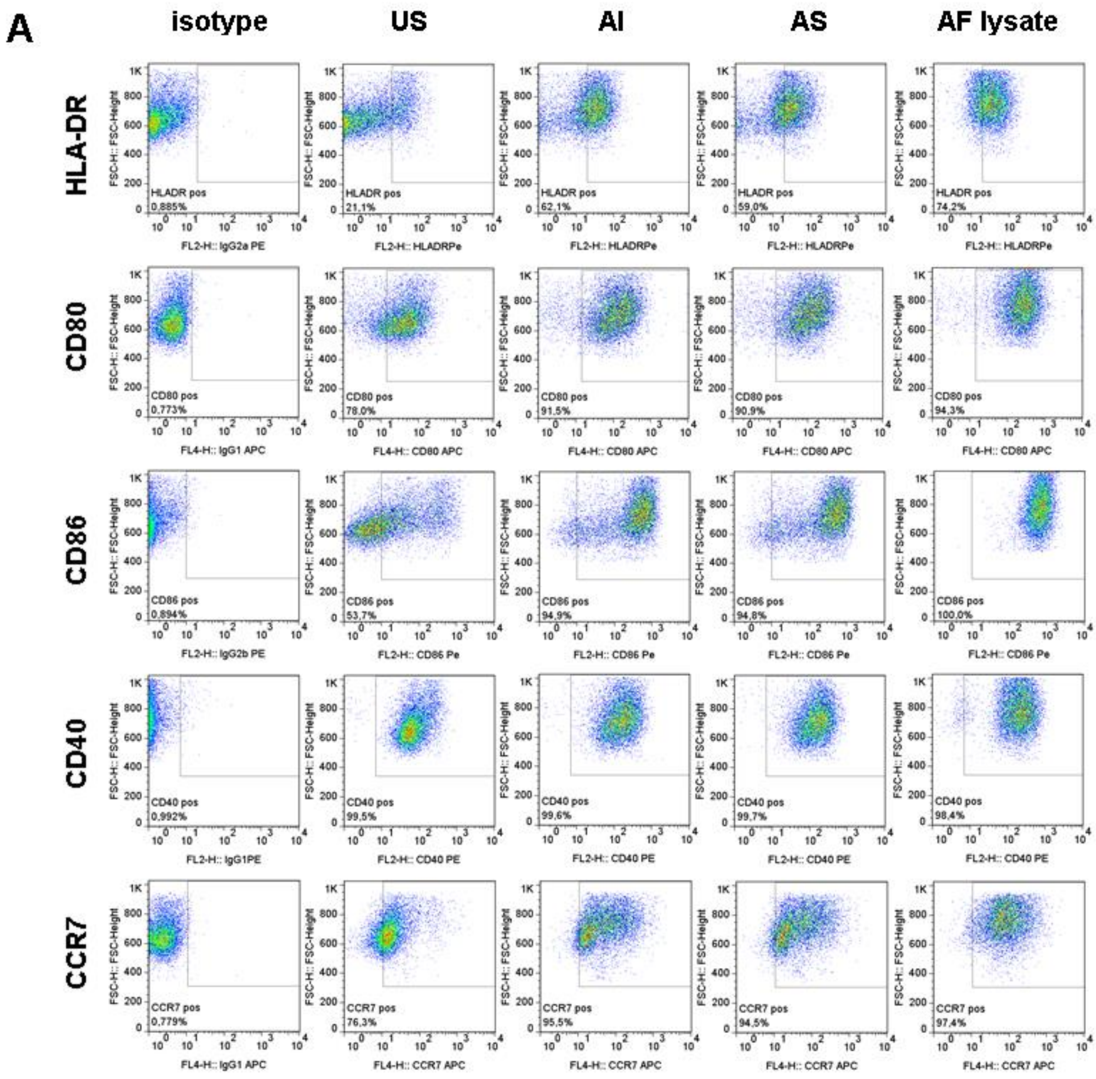

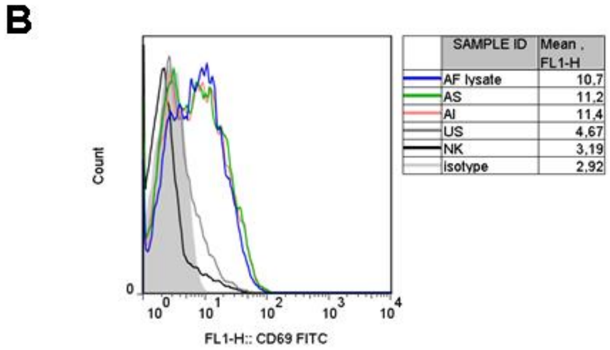

***A. fumigatus* stimulated DCs can activate autologous, resting NK cells.** (A) DCs were either left untreated (US) or were stimulated with soluble (AS, 10 µg/ml) and insoluble (AI, 5 µg/ml) cell wall fractions or *A. fumigatus* lysate (AF lysate, 5 µg/ml) for 24 h. Data display percent positive cells of the maturation markers HLA-DR, CD80, CD86, CD40 and CCR7 or perspective isotype controls. (B) NK cells were left untreated (NK) or co-cultured with previously AI, AS, AF lysate stimulated or unstimulated DCs (US). NK cell activation was measured by the mean fluorescence intensities of anti-CD69 or isotype control stained NK cells. Data are representatives of three independent experiments.

Supplementary Figure 4:

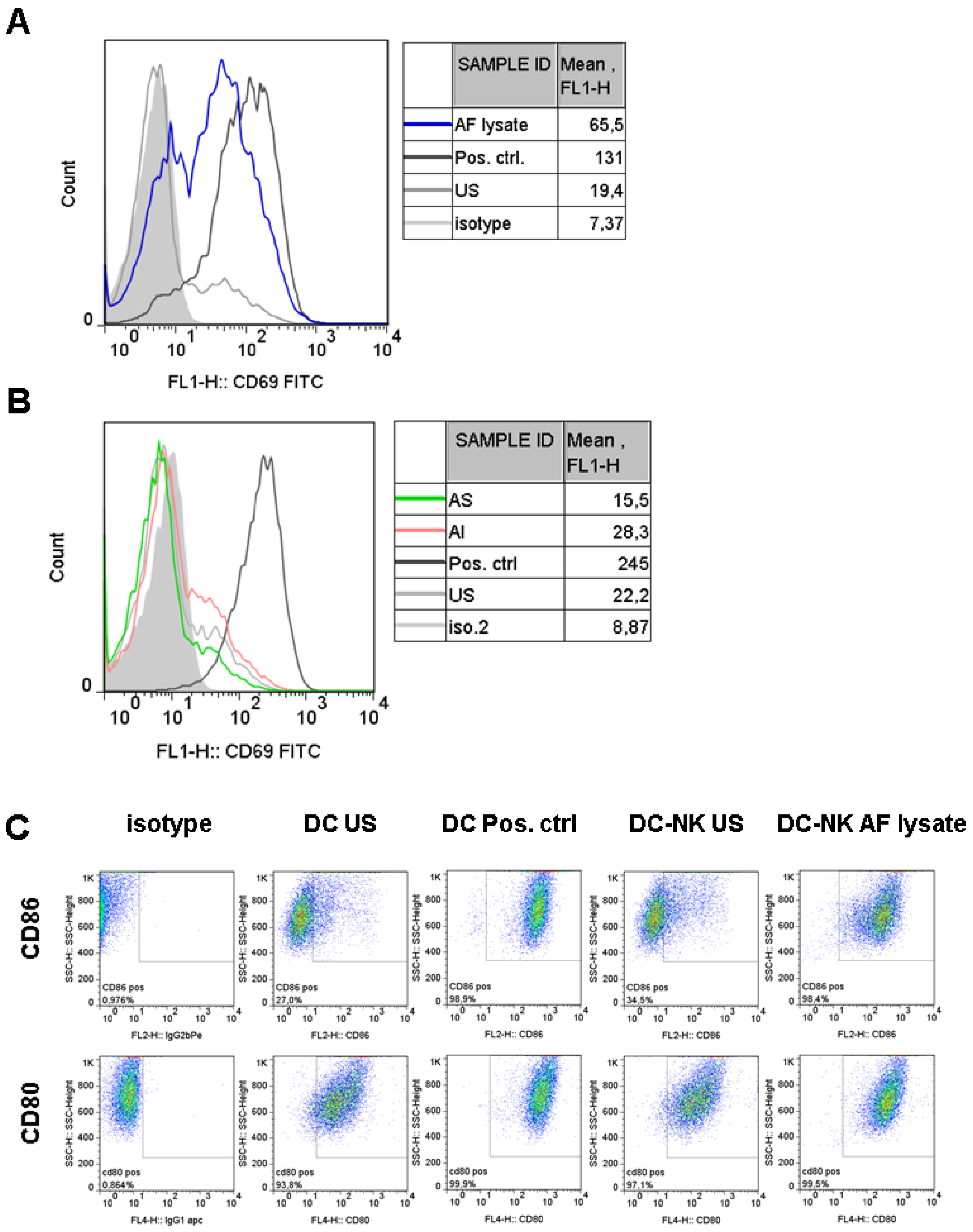

NK cell activation after stimulation with fungal components and NK – DC co-culture. (A, B) NK cells were either left untreated (US) or were stimulated with *A. fumigatus* lysate (AF lysate, 1 µg/ml), positive control (Pos. ctrl., 500 U/ml IL-15), alkali insoluble cell wall fraction (AI, 5 µg/ml) or alkali soluble cell wall fraction (AS, 10 µg/ml) for 24 h. NK cells were stained with anti-CD69 antibody or perspective isotype control and data are displayed as mean fluorescence intensity (MFI). (C) DCs were either left untreated (DC US) or were co-cultured with autologous, unstimulated (DC-NK US) or pre-stimulated (DC-NK AF lysate) NK cells for 16 h. As a positive control, DCs were stimulated with 1 µg/ml LPS (DC Pos. ctrl). DC maturation was analyzed by CD80 and CD86 positivity. Data are representatives of three independent experiments.

Supplementary Figure 5:

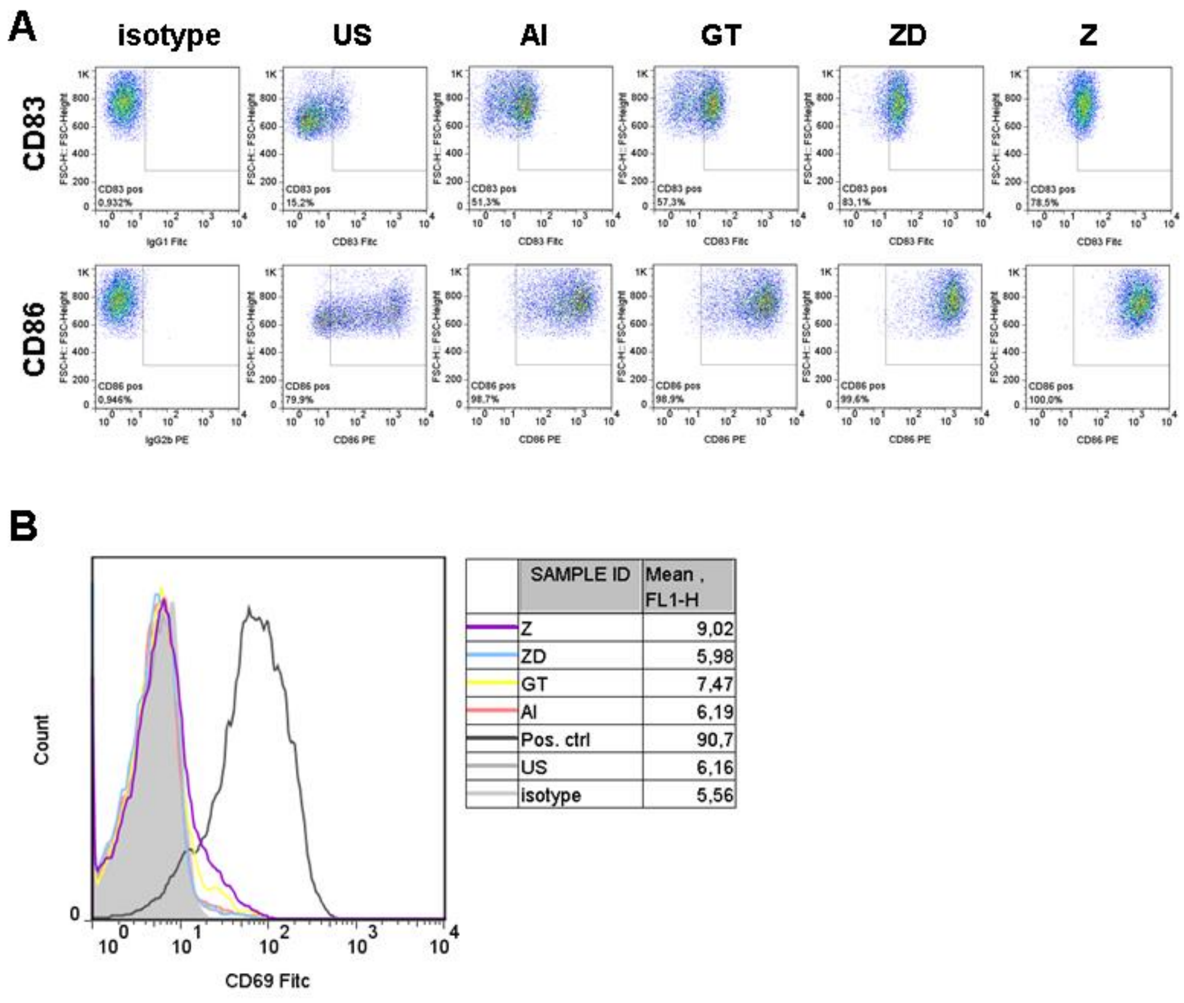

Stimulation of DCs and NK cells with cell wall fraction, inactivated germ tubes, zymosan depleted and zymosan. (A) DCs were left untreated (US) or were stimulated with alkali insoluble cell wall fraction (AI, 5 µg/ml), inactivated *A. fumgiatus* germ tubes (GT, MOI 1), zymosan depleted (ZD, 100 µg/ml), or zymosan (Z, 10 µg/ml) for 9 h. DC maturation was analyzed by CD83 and CD86 positive cells. As a control, cells were stained with the perspective isotype control antibodies. (B) NK cells were left untreated (US) or were stimulated with either the positive control (Pos. ctrl, IL-15, 500 U/ml), AI (5 µg/ml), GT (MOI 1), ZD (100 µg/ml), or Z (10 µg/ml) for 16 h. NK cell activation was investigated by staining with anti-CD69 antibody or isotype control. Data are displayed as mean fluorescence intensities (MFI) and are representatives of three independent experiments.

Supplementary Figure 6:

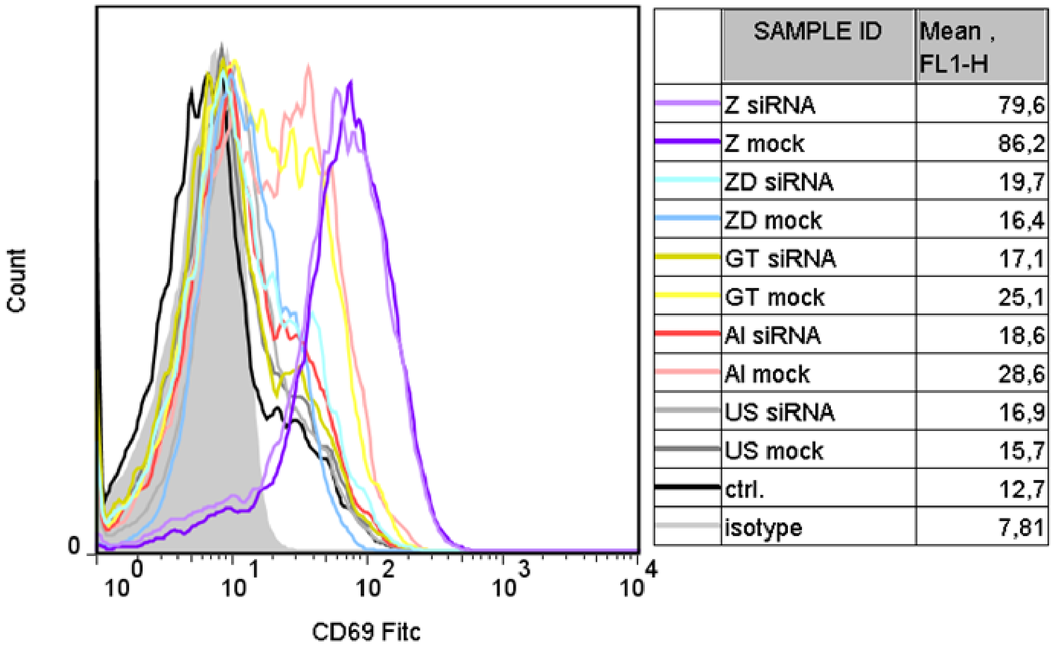

Soluble factors derived from Dectin-1 silenced and mock silenced DCs activate autologous NK cells. Dectin-1 silenced (siRNA) or mock silenced (mock) DCs were stimulated with the alkali insoluble cell wall fraction (AI, 5 µg/ml), inactivated *A. fumigatus* germ tubes (GT, MOI 1), zymosan depleted (ZD, 100 µg/ml), zymosan (Z, 10 µg/ml), or were left untreated (US) for 9 h. Soluble factors from DC stimulation were transferred onto resting, autologous NK cells for 16 h. NK cells were stained with anti-CD69 antibody or isotype control and data are displayed by mean fluorescence intensity (MFI). Data are representatives of five independent experiments.

Supplementary Figure 7:

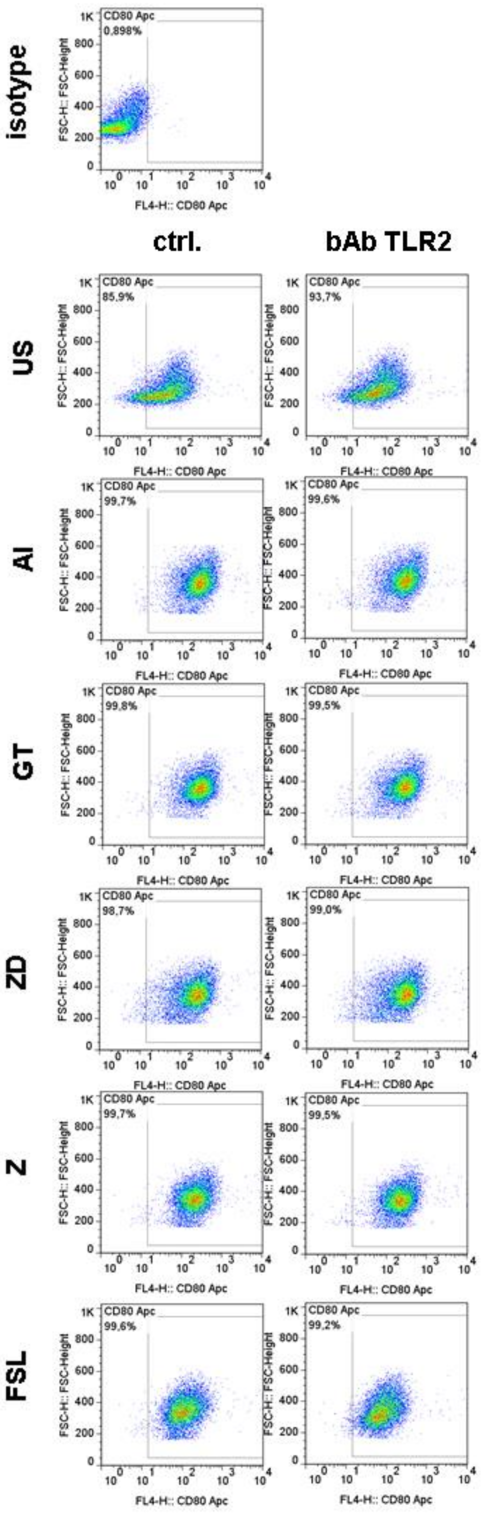

TLR2 blocking and DC stimulation. DCs were treated with TLR2 blocking antibody (bAb, 10 µg/ml) or isotype control (ctrl.) for 1 h. DCs were diluted with medium and stimulated with the alkali insoluble cell wall fraction (AI, 5 µg/ml), inactivated *A. fumigatus* germ tubes (GT, MOI 1), zymosan depleted (ZD, 100 µg/ml), zymosan (Z, 10 µg/ml), FSL (100 ng/ml) or were left untreated (US) for 24 h. Cells were stained with anti-CD80 antibody or isotype control and data show representatives of five independent experiments.

Supplementary Figure 8:

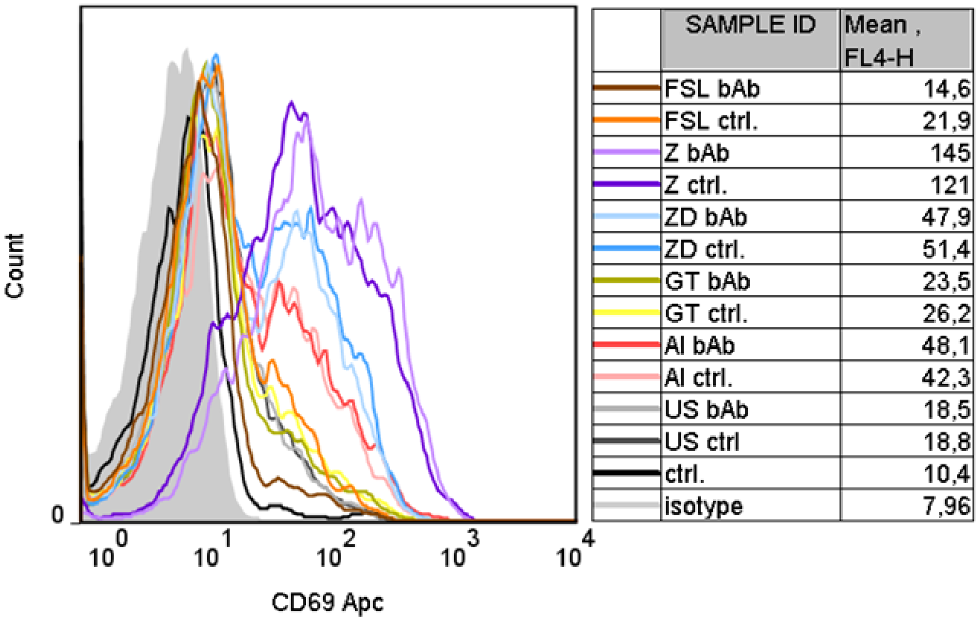

	SAMPLE ID	Mean , FL4-H
	FSL bAb	14,6
	FSL ctrl.	21,9
	Z bAb	145
	Z ctrl.	121
	ZD bAb	47,9
	ZD ctrl.	51,4
	GT bAb	23,5
	GT ctrl.	26,2
	AI bAb	48,1
	AI ctrl.	42,3
	US bAb	18,5
	US ctrl	18,8
	ctrl.	10,4
	isotype	7,96

Soluble factors derived from TLR2 blocked DCs activate autologous NK cells. DCs treated with TLR2 blocking antibody (bAb, 10 µg/ml) or isotype control (ctrl) were stimulated with the alkali insoluble cell wall fraction (AI, 5 µg/ml), inactivated *A. fumigatus* germ tubes (GT, MOI 1), zymosan depleted (ZD, 100 µg/ml), zymosan (Z, 10 µg/ml), FSL (100 ng/ml) or were left untreated (US) for 24 h. Soluble factors from DC stimulation were transferred onto resting, autologous NK cells for 16 h. NK cells were stained with anti-CD69 antibody or isotype control and data are representatives of four independent experiments.

Supplementary Figure 9:

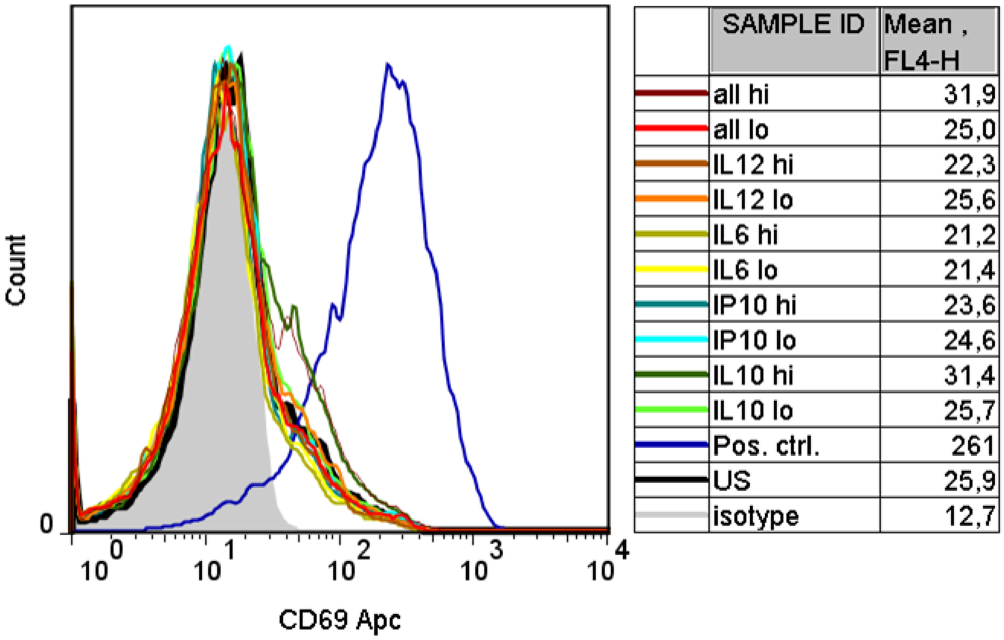

NK cell stimulation with cytokines and chemokines. NK cells were isolated from thawed PBMCs and were stimulated with IL-6 (10 ng/ml and 50 ng/ml), IL-10 (1 ng/ml and 5 ng/ml), IP-10 (4 ng/ml and 5 ng/ml), IL-12 (5 ng/ml and 10 ng/ml) or the combination of all cytokines and chemokines in low (lo) or high (hi) concentration, respectively. After 16 h, NK cell activation was analyzed by staining with anti-CD69 antibody or isotype control. Data are displayed as mean fluorescence intensities and are representatives from one of three independent experiments.

Supplementary Figure 10:

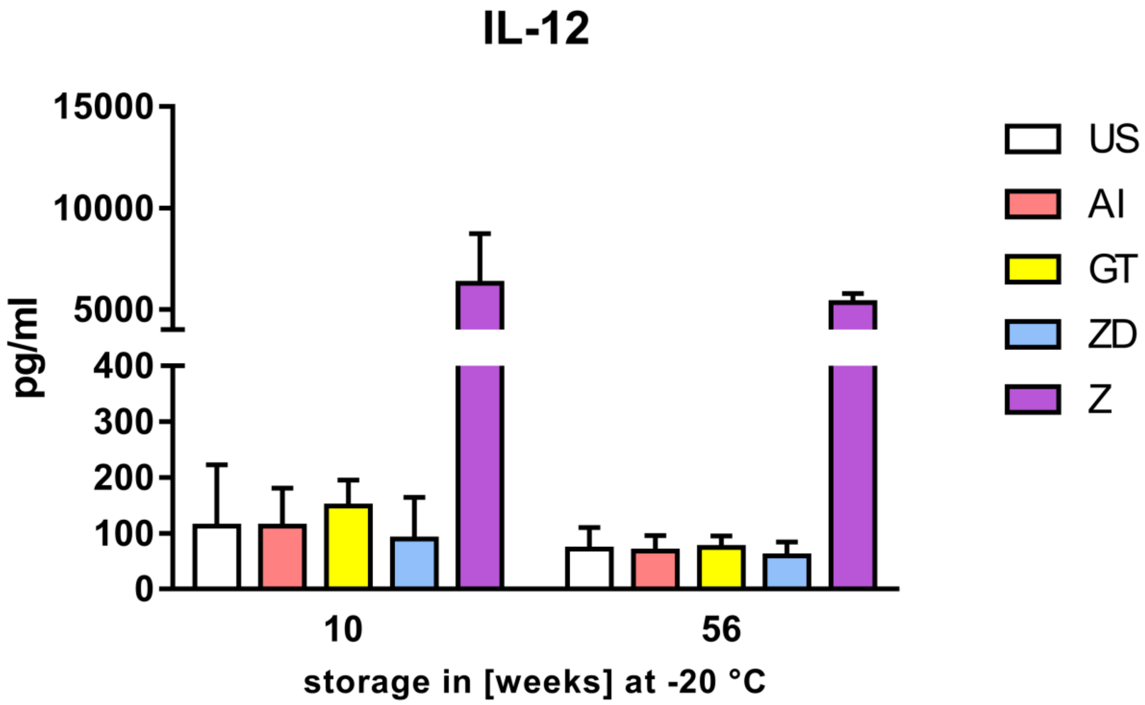

Storage of supernatants at – 20 °C. DCs were stimulated with the alkali insoluble cell wall fraction (AI, 5 µg/ml), inactivated *A. fumigatus* germ tubes (GT, MOI 1), zymosan depleted (ZD, 100 µg/ml), zymosan (Z, 10 µg/ml), or were left untreated (US) for 9 h. Supernatants were frozen at – 20 °C and were analyzed by multiplex immunoassay 10 weeks after sample collection. Samples were frozen again and were analyzed by IL-12 ELISA 56 weeks after sample collection. Data are represented as mean + SEM of n = 3 independent experiments. There were no significant differences found when results (10 vs. 56 weeks of storage) were compared by two-way ANOVA.

Supplementary Figure 11:

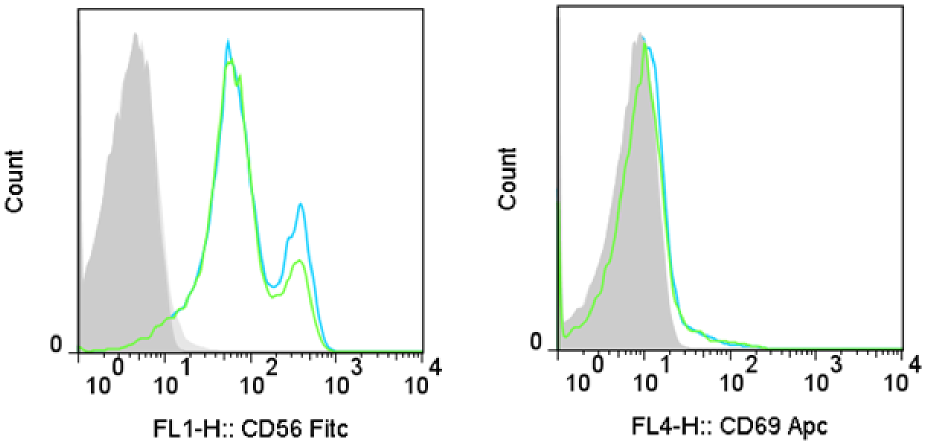

	SAMPLE ID	Mean , FL1-H
	sample w/o FcR block	109
	isotype w/o FcR block	4,30
	sample + FcR block	131
	isotype + FcR block	4,77

	SAMPLE ID	Mean , FL4-H
	sample w/o FcR block	12,6
	isotype w/o FcR block	8,72
	sample + FcR block	12,4
	isotype + FcR block	7,72

Flow cytometric analyses with and without FcR blocking reagent. NK cells were isolated from thawed PBMCs and were stained in a total volume of 100 µl cell suspension in HBSS + 1 % FCS + 0.4 % EDTA including 20 % FcR blocking reagent (Miltenyi Biotec) or without FcR blocking reagent. Cells were stained with anti-CD56 (FITC), anti-NKp46 (PE), anti-CD3 (PerCP) and anti-CD69 (APC) antibodies for 20 min at 4 °C. Then, unbound antibodies were washed away and samples were analyzed by flow cytometry. NK cells were gated into NKp46+CD3- cells before CD56 and CD69 fluorescence was further investigated. Data are representatives of n = 3 independent experiments.

Supplementary Figure 12:

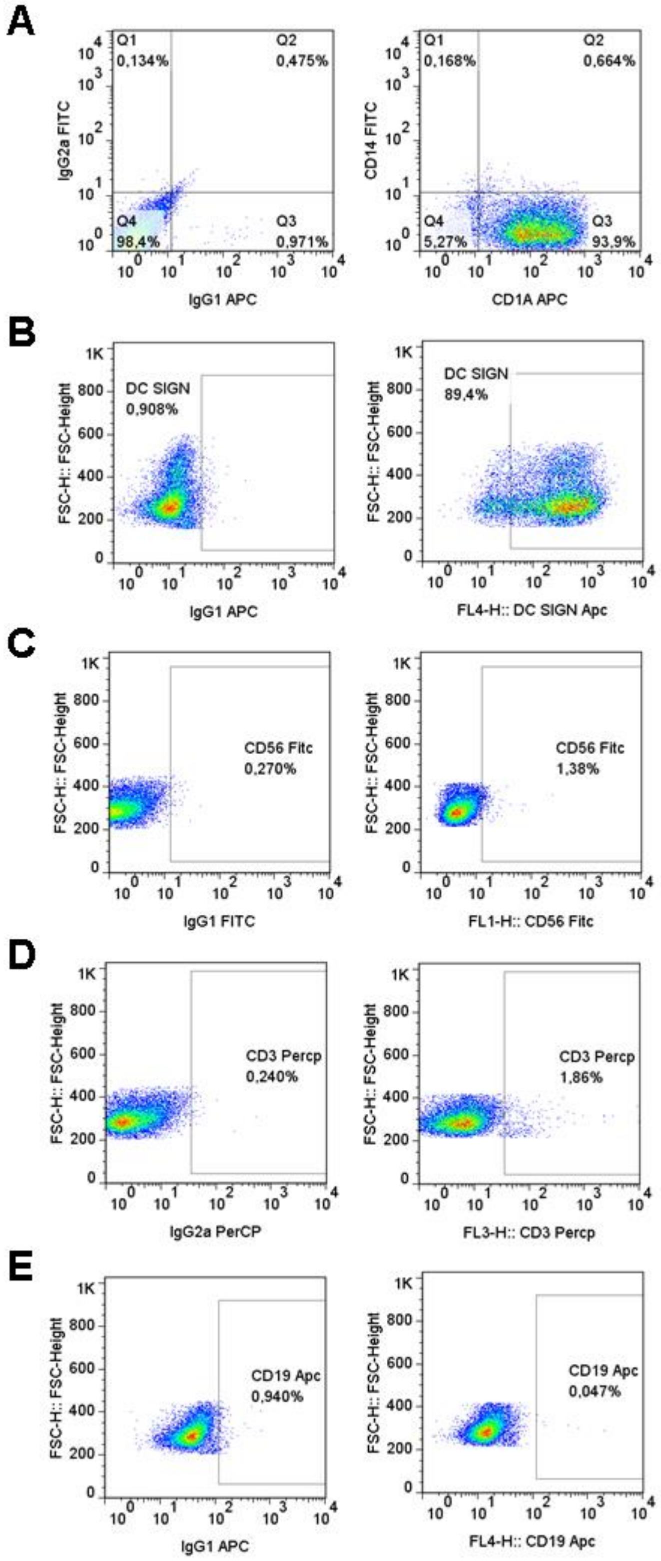

moDC purity. Monocytes were cultured (2.5 x 10^6/3 ml) in 6-well plates for 5 days with the addition of IL-4 and GM-CSF. Cells were analyzed regarding the expression of (A) CD1a and CD14, (B) DC-SIGN, (C) CD56, (D) CD3 and (E) CD19. Data are representatives of n = 15 (A), n = 2 (B-E) independent experiments.
